# Bio-impedance measurement allows displaying the early stages of neutrophil extracellular traps

**DOI:** 10.17179/excli2020-2868

**Published:** 2020-11-05

**Authors:** Caren Linnemann, Sascha Venturelli, Franziska Konrad, Andreas K. Nussler, Sabrina Ehnert

**Affiliations:** 1 Siegfried Weller Institute for Trauma Research, BG Unfallklinik Tuebingen, Eberhard Karls Universität Tuebingen, Tuebingen, Germany; 2 Institute of Physiology, Department of Vegetative and Clinical Physiology, University Hospital Tuebingen, Tuebingen, Germany; 3 Institute of Nutritional Sciences, Department of Nutritional Biochemistry, University of Hohenheim, Stuttgart, Germany; 4 Department of Anesthesiology and Intensive Care Medicine, University Hospital of Tuebingen, Tuebingen, Germany

**Keywords:** bio-impedance, neutrophils, neutrophil extracellular traps, Sytox Green Assay, xCelligence, immune cell activation

## Abstract

Neutrophils are the most abundant immune cells in the blood. Besides common immune defense mechanisms, releasing their DNA covered with antimicrobial proteases and histones represent another strong defense mechanism: neutrophil extracellular traps. *In vitro* the two most common inducers of these, so called, NETs are calcium ionophores (CI) and PMA (Phorbol 12-myristate 13-acetate). Following stimulation monitoring of NET release is necessary. For now, the methods of choice are quantification of free DNA by fluorescent dyes or analysis of immunofluorescence images. As a new method we tested bio-impedance monitoring of neutrophils after stimulation with the two inducers PMA and CI in gold-electrode coated plates. Bio-impedance (cell index) was measured over time. Results were compared to the monitoring of NETs by the fluorescent DNA-binding dye Sytox Green and immunofluorescence analysis. Cell index peaked about 25 min faster following CI stimulation than following PMA stimulation. The activation in Sytox Green Assay was significantly later detectable for PMA (+ approx. 90 min) but not for CI stimulation. The earlier and faster activation by CI was also confirmed by immunofluorescence staining. Our data suggest that bio-impedance measurement allows an easy online tracking of early neutrophil activation. This offers new opportunities to monitor early phases and stimuli-dependent dynamics of NETosis.

## Abbreviations

**CI,** calcium ionophore A23187; **PMA,** Phorbol 12-myristate 13-acetate; **PBMCs**, peripheral blood mononuclear cells; **MPO**, myeloperoxidase; **NE**, neutrophil elastase; **NETs**, neutrophil extracellular traps; **RTCA**, real time cell analysis; **PMN**, polymorphonuclear cells; **ECIS**, electric cell-substrate impedance sensing

## Introduction

Neutrophils are one of the main players of the innate immune response. They are the first cells to be recruited to inflammation and infection sites and can release large amounts of cytokines. They attract further immune cells and can phagocytose high amounts of pathogens (Amulic et al., 2012[1]; Nordenfelt and Tapper, 2011[37]). But pathogens can also be tackled by another mechanism: formation of neutrophil extracellular traps (NETs) (Brinkmann et al., 2004[7]). NETs are large structures composed of decondensed DNA with proteins coupled to it. The sticky DNA and the coupled defense proteins like myeloperoxidase (MPO), neutrophil elastase (NE), cathepsins, and other proteases allow trapping of pathogens and the destruction of those. They are especially effective against large pathogens and those who can evade phagocytosis (Rohm et al., 2014[44]; von Köckritz-Blickwede et al., 2016[51]). The composition of the associated proteins can vary but some basic proteins (myeloperoxidase, cathepsin G, histones, granzyme G, cathelicidin, catalase) are proposed to build the basis of the released proteins (Bruschi et al., 2019[8]; Petretto et al., 2019[40]).

Like with many other potent immune defense mechanisms, NETs can harm tissue when overactivated, and have been associated with many diseases in the last years such as cancer and metastasis (Cools-Lartigue et al., 2013[11]; Tohme et al., 2016[49]), thrombosis (Martinod et al., 2013[31]; Perdomo et al., 2019[38]), and atherosclerosis (Liu et al., 2018[29]; Warnatsch et al., 2016[53]). Very recently a role of neutrophils and their released NETs has been found in severe courses of COVID-19 (Leppkes et al., 2020[27]; Middleton et al., 2020[34]; Wang et al., 2020[52]) suggested to be one of the causal factors of thrombotic events. Briefly, NETs seem to be significantly involved in many inflammatory processes where neutrophils are participating.

In order to investigate NETs in various pathologies, it is urgently needed to have easy applicable tools to measure the fast dynamics of NET formation, as well as to do detailed analysis and quantification. The most detailed analysis is possibly by imaging neutrophils and their released NETs by either electron microscopy or immunofluorescence analysis (Giaglis et al., 2016[17]; Remijsen et al., 2011[43]; Wu et al., 2019[54]) but these methods do not allow screening of stimuli or fast multiple-time point measurements. 

Therefore, “Sytox Green Assay” was developed as a fast and easy method to monitor DNA release from neutrophils (Langsrud and Sundheim, 1996[25]). Sytox Green shows up to 500-fold increase in fluorescence upon binding to DNA (Roth et al., 1997[45]) and allows screening of many different stimuli but without strong specificity.

Another simple method for NET quantification is to measure the DNA release into culture supernatants by staining DNA with a DNA-binding fluorescent dye (Munoz-Caro et al., 2015[35]). Such a method is also used to analyze cell-free DNA in patient samples like sera or tissues (Arai et al., 2013[2]; Lee et al., 2018[26]). Similarly, the number of protein-DNA complexes in fluids can be determined (MPO-DNA, NE-DNA) by ELISA which seems to be more specific than just the determination of free DNA in supernatants (Kano et al., 2017[21]). 

Bio-impedance is commonly used to measure body composition in all-day situations and also in larger health studies (Brantlov et al., 2019[5]; Li et al., 2013[28]). The method relies on the dielectric behavior of cells (Giaever and Keese, 1993[16]) and allows easy and non-invasive measurements of parameters like cell-free mass or fat fraction. To analyze cell behavior by bio-impedance *in vitro*, the first commercially available instrument was launched in 2004 (https://www.aceabio.com/about/, May, 07, 2020). However, Giaever and Keese described the first plates with gold electrode coating already in 1993 (Giaever and Keese, 1993[16]). The gold coated plates allowed easy measurements of cell alterations such as cell growth, detachment, or attachment (Xiao et al., 2002[55]). Depending on the used frequencies different cell behaviors are more or less in focus (Schröter et al., 2015[46]).

Nowadays, bio-impedance is an established method to monitor cell killing (Seidel et al., 2014[48]), the activation of immune cells (T-cells (Guan et al., 2013[18]), natural killer cells (Fasbender and Watzl, 2018[14])) and the interaction of different cell types (Gagliardi et al., 2015[15]; Ludwig et al., 2011[30]; Yang et al., 2019[56]). For some applications like cell migration special devices have been developed based on a modified version of classical assays like the Boyden chamber assay (xCelligence® real-time cell analysis (RTCA) DP system (Preuss et al., 2014[42])). One of the biggest advantages is the online measurement of cell behavior. 

The xCelligence systems use a parameter called cell index as their readout: the device normalizes the measured impedance to a defined value, normally the background value. Commonly used is also the normalized cell index where the measured values are normalized to a certain value (*e.g.,* value at the time point of the application of a drug) (Kho et al., 2015[23]). Very dense cellular layers result in an increased cell index by impairing the bio-electrical impedance. Thus the cell index is influenced by cellular morphology, density, and cell-substrate interaction (Arndt et al., 2004[3]; Vistejnova et al., 2009[50]; Yu et al., 2018[57]). 

To measure activation of neutrophils by bio-impedance measurement is uncommon. Bio-impedance was used to measure neutrophil attachment or neutrophil chemotaxis (Cano et al., 2016[9]; Yu et al., 2018[57]). A biosensor for the rapid testing of activation and sorting of neutrophils based on an impedance cytometer was recently developed (Petchakup et al., 2019[39]). Mathematical modeling of the bio-impedance of NET formation was made (Schröter et al., 2015[46]), and an applicator for wounds was developed (Schröter et al., 2013[47]). However, the use of bio-impedance measurements for the systematic research of NETs *in vitro* has not yet been reported.

This work presents the possibility of measuring the bioelectrical impedance with the xCelligence systems to measure NET formation in combination with the imaging of living cells. We compared this assay with the established methods of the Sytox Green Assay and immunofluorescence analysis.

## Material and Methods

### Neutrophil isolation

Neutrophils were isolated freshly from blood taken from 15 healthy volunteers. Venous blood was taken with a butterfly needle into EDTA-tubes (S-Monovette 9 mL, Sarstedt, Germany) and directly used for density gradient centrifugation. 6 mL blood was carefully layered on 6 mL of Lympholyte poly cell separation medium (Cedarlane, Burlington, Ontario, Canada). Samples were centrifuged for 35 min at 500 g without break at room temperature. The plasma and peripheral blood mononuclear cell (PBMC) layers were discarded and the polymorphonuclear cell (PMN) layer carefully taken with a pipette and transferred to a 15 mL tube. PMN layer was washed twice with PBS (12 mL, centrifugation at 450 g, 10 min, room temperature without break) and taken up in RPMI medium (RPMI-1640 without phenol red, Sigma-Aldrich, Munich, Germany). Cells were counted by Trypan Blue exclusion method in a Neubauer counting chamber, without counting of residual erythrocytes. Cells were prepared to a density of 1x10^6^ cells/mL.

### Stimulation of neutrophils

Neutrophils were stimulated with 100 nM PMA (Phorbol 12-myristate 13-acetate, Abcam, Cambridge, UK) or 4 µM Calcium ionophore A23187 (CI, Sigma-Aldrich, Munich, Germany). Sytox Green was added to reach a final concentration of 1 µM (Thermo Fisher Scientific, Karlsruhe, Germany). Stimulation solutions were prepared in 1.25-fold of final concentration to allow measurement of blank (80 µL) and thereafter addition of concentrated cell suspension (20 µL).

### Sytox Green Assay

80 µL Sytox Green containing medium with or without stimuli were added to a 96-well plate (Greiner Bio-One, Frickenhausen, Germany). 20 µL of neutrophil suspension was added to receive a final concentration of 2x10^5^ cells/mL. Measurements were done in triplicates. To improve comparability between donors, cells were also lysed with 1 % Triton-X-100 (Carl Roth, Karlsruhe, Germany) which allows normalization to total DNA. Fluorescence was measured every 30 min in an Omega Plate Reader (BMG Labtech, Ortenberg, Germany) at 485 nm/520 nm. At 3 h timepoint microscopy pictures of the images were taken in the GFP channel with an Evos FL imaging system (Thermo Fisher Scientific).

### xCelligence measurement

The xCelligence RTCA eSsight device (Omni Life Sciences, OLS, Bremen, Germany) was used to measure bio-impedance. It was placed into an incubator at 37 °C at 5 % CO_2_ in a humidified atmosphere. 

The device uses electric cell-substrate impedance sensing (ECIS) to monitor current changes for the calculation of bio-impedance values. Similar to the Sytox Green Assay, 80 µL of the prepared stimulation solutions were added to a 96-well xCelligence measurement plate (OLS) in quadruplicates. Then, the plate was measured in the RTCA eSight device (OLS) as blank. Afterwards, plates were transferred to a sterile bench and 20 µL of the isolated neutrophils were added to reach a final concentration of 2x10^5^ cells/mL. For a period of 6 h cell index was measured at least every 15 min (run one every 15 min, run two every 5 min). Fluorescence pictures were taken every 30 min (run one, 4 images/well, bright field and green fluorescence) or every 15 min (run two, 2 images/well). Data were exported as Excel files, manually normalized and, XY diagrams made with Graphpad Prism Version 8.0. Peak times of PMA stimulation were determined by partial curve fitting *via* a quadratic function (Y=B_0_ + B_1_X + B_2_X^2^) and determination of the peak point by the first derivative. Peak times of control cells and CI stimulation were analyzed manually (time point of highest measured value apart from start value) from the measured data.

Fluorescence pictures from live cell imaging were exported as single tiff images and combined manually with ImageJ Version 2.0 (NIH, Bethesda, Maryland, USA). For quantification, images of the time course (one image/well of GFP channel) were subjected to automatic counting by Image J. Therefore, auto local threshold was set (Bernsen, radius 30) and watershed separation was done of the binary images. Particles acceding a size of 1000 pixel^2^ were counted automatically.

### Immunofluorescence

To obtain a time-course analysis of stimulation with PMA and calcium ionophore Ibidi µ-slides (8-well, Ibidi, Martinsried, Germany) were coated with a 1:5 dilution of Poly-L-Lysine (Sigma-Aldrich), washed once with sterile water and air-dried. Neutrophils of 4 donors were isolated and seeded onto the µ-slides in a concentration of 3x10^5^ cells/mL in plain RPMI 1640 medium (Sigma-Aldrich). Cells were stimulated with 100 nM PMA or 4 µM calcium ionophore. At each time point (1-6 h, every hour) cells were fixed with 4 % formaldehyde for 30 min, and afterwards permeabilized with 0.5 % Triton-X-100 for 5 min. Wells were blocked with 5 % BSA (AppliChem, Darmstadt, Germany) for 1 h and stained overnight with anti-myeloperoxidase antibody in 1:200 dilution in PBS (sc-52707, Santa Cruz Biotechnology, Heidelberg, Germany). Slides were washed 3 times with PBS for 10 min. Staining was completed by a 2 h incubation with Alexa Fluor 488 coupled secondary anti-mouse antibody (1:1000 in PBS, Invitrogen, Thermo Fisher Scientific) and Hoechst 33342 nuclear counterstaining (2 ng/µL in PBS, Thermo Fisher Scientific). After three times washing for 10 min with PBS five images per condition were taken in 10-fold original magnification.

### Immunofluorescence analysis

Immunofluorescent images were analyzed with ImageJ using a method adapted from Brinkmann et al. (2012[6]). From Hoechst 33342 staining (DAPI channel) all nuclei were automatically counted after threshold setting. From GFP channel (myeloperoxidase staining) only cells that exceed a certain size and are not round were counted automatically after threshold setting. The threshold was adapted to unstimulated cells so that nearly no counting occurred for not activated cells. By building the ratio of MPO (myeloperoxidase) to Hoechst staining the proportion of activated cells was calculated. As a second readout, the nuclear size was determined from Hoechst 33342 staining (bigger size means stronger activation).

### Statistical analysis

If not stated differently data are shown as box plots “Tuckey” with median, interquartile range, and 95 % confidence interval. Kruskal-Wallis test followed by Dunn's correction for multiple comparisons was done for testing of significance. A p-value below 0.05 was considered significant.

## Results

### Analysis of activation by Sytox Green Assay

The dynamics of DNA release from stimulated neutrophils was assessed by Sytox Green Assay. Neutrophils were stained with 1 µM Sytox Green and stimulated with PMA or calcium ionophore and fluorescence measured for 5 h. 

**Figure 1:**
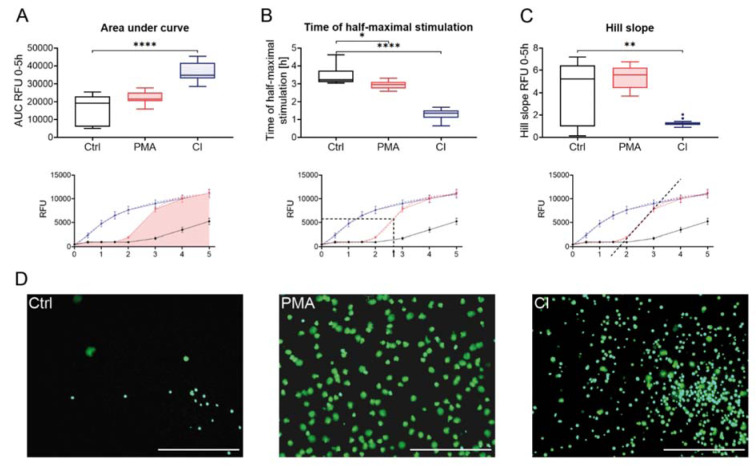
Sytox Green Assay of stimulated neutrophils. Neutrophils were stained with 1 µM Sytox Green and fluorescence measured over a period of 5 h. Different analysis methods were used to show neutrophil activation by different stimuli: (A) Analysis of DNA release by measurement of area under curve; (B) Analysis of stimulation speed by determination of the time when half-maximal DNA-release was reached; (C) Analysis of dynamics of stimulation by measurement of hill slope. Lower panels illustrate the analyses graphically, dashed lines indicate interpolated curves. (D) Microscopy pictures of neutrophils in green channel (Sytox Green signal). Scale bar = 400 µm. N=15, n=3. Statistical analysis was done by Kruskal-Wallis test followed by Dunn's correction for multiple comparisons. *p<0.05; ** p<0.01; ****p<0.0001. Ctrl: Control, PMA: phorbol 12-myristate 13-acetate, CI: calcium ionophore A23187, AUC: Area under curve, RFU: relative fluorescence unit.

Three different readouts were used to display the release of DNA: Area under curve (Figure 1A(Fig. 1)), analysis of the half-maximal time of stimulation (Figure 1B(Fig. 1)), and hill slope analysis (Figure 1C(Fig. 1)). The area under curve analysis revealed that stimulation with calcium ionophore resulted in the highest amount of DNA released over time. Stimulation with PMA also showed a higher area under curve value than the control, but not significant. The time of half-maximal stimulation (Figure 1B(Fig. 1)), representing the speed of the reaction, was the lowest for the stimulation with CI (1.32 ± 0.27 h). Cells stimulated with PMA took about double the time to reach half-maximal stimulation (2.95 ± 0.21 h). After a certain time (4-5 h) the values for the control cells also increased. The hill slope value provides information about the dynamics of the stimulation. For stimulation with calcium ionophore, the hill slopes were low because the increase in released DNA is not as fast as for stimulation with PMA, where the hill slopes were very high due to the fast release of DNA after a certain time point. Control cells had a hill slope in a similar range to PMA stimulated cells since cells from some donors were activated quite fast without stimulation. In general, the values show high variation between the donors. Figure 1D(Fig. 1) shows microscopic images after 3 h of stimulation of an exemplary donor. To reduce the effects of donor variability fluorescence values can be normalized to the value of total DNA (Triton-treated cells, Supplementary Figure 1).

### Analysis of neutrophil activation by bio-impedance measurement

**Figure 2:**
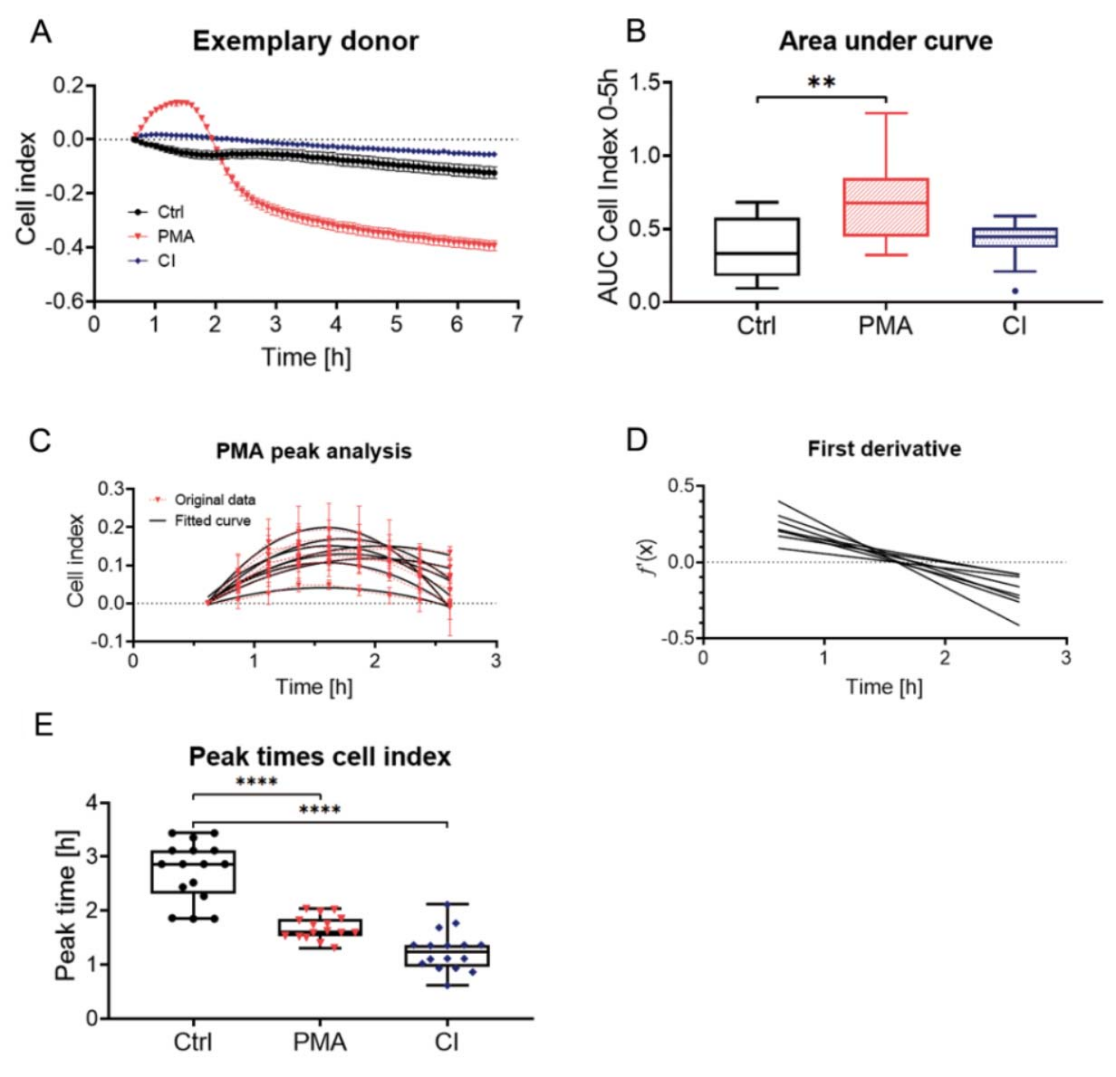
Bio-impedance measurement for monitoring of activation of neutrophils. Neutrophils were seeded in eSight E-Plate VIEW 96 and bio-impedance was measured for a period of 6 h. (A) shows measurement of an exemplary donor for ctrl, as well as PMA and CI stimulated cells (baseline set to 30 min). (B) Analysis of cell index by area under curve measurement, N=15. (C) Analysis of time point of PMA peak by partial curve fitting of peaks of 8 single donors with originally measured data (red, dashed line) and fitted curves (black). (D) First derivate curves calculated from the fitted curves of (C) which allows calculation of (E) peak time of PMA stimulation by determination of x-intercept for the different donors. (E) Peak times for the different donors for Ctrl and CI stimulation (Manual determination from highest measured value) and PMA stimulation (determination by quadratic curve fit). N = 15, n = 4. Statistical analysis was done by Kruskal-Wallis test followed by Dunn's correction for multiple comparisons. ** p<0.01, ****p<0.0001. Ctrl: Control, PMA: phorbol 12-myristate 13-acetate, CI: calcium ionophore A23187, AUC: Area under curve.

The activation dynamics were determined based on changes in bio-impedance. As some donors showed a sharp drop at the beginning of the measurement (Supplementary Figure 2) the baseline was set to the 30 min value. For control cells, bio-impedance was reduced over time compared to the basal level with a small peak between 2-4 h (Figure 2A(Fig. 2)). PMA and CI stimulation both showed a stronger peak in the cell index (Figure 2A(Fig. 2)). For PMA stimulation this peak was much higher than for CI stimulation, where the peak came earlier and was much flatter. Figure 2B(Fig. 2) gives an overview of the stimulation of all 15 donors by area under curve analysis. PMA had a significantly higher area under the curve than CI or Ctrl. CI showed no significant difference to Ctrl which can be explained by the flat peak and fast stimulation by CI. 

For both kinds of stimulation (PMA and CI) and the Ctrl, the peak time was analyzed. For PMA stimulation partial curve fitting with a quadratic term (Figure 2C(Fig. 2)) and determination of the local maximum from the first derivative was possible (Figure 2D(Fig. 2)). The quality of curve fit can be found in Supplementary Table 1. For PMA the mean peak time was determined with 1.68 ± 0.22 h. For calcium ionophore and the control, curve fitting was not possible, and the peak time was determined manually with a mean of 1.26 ± 0.38 h for CI and 2.74 ± 0.55 h for the control (Figure 2E(Fig. 2)). Figure 2E(Fig. 2) summarizes the peak times for Ctrl, as well as PMA and CI stimulation with single dots indicating the single donors. It shows that the Ctrl peak time is significantly later compared to PMA or CI stimulation. Compared to the half-maximal stimulation of the Sytox Green Assay the times for calcium ionophore stimulation were comparable (Sytox Green Assay 1.32 ± 0.27 h, *p*>0.99) whereas for PMA stimulation the half-maximal stimulation time in the Sytox Green Assay was much later (2.95 ± 0.21 h, *p*=0.0076).

### Live fluorescent imaging allows quantification and visualization of NET release in a single step

**Figure 3:**
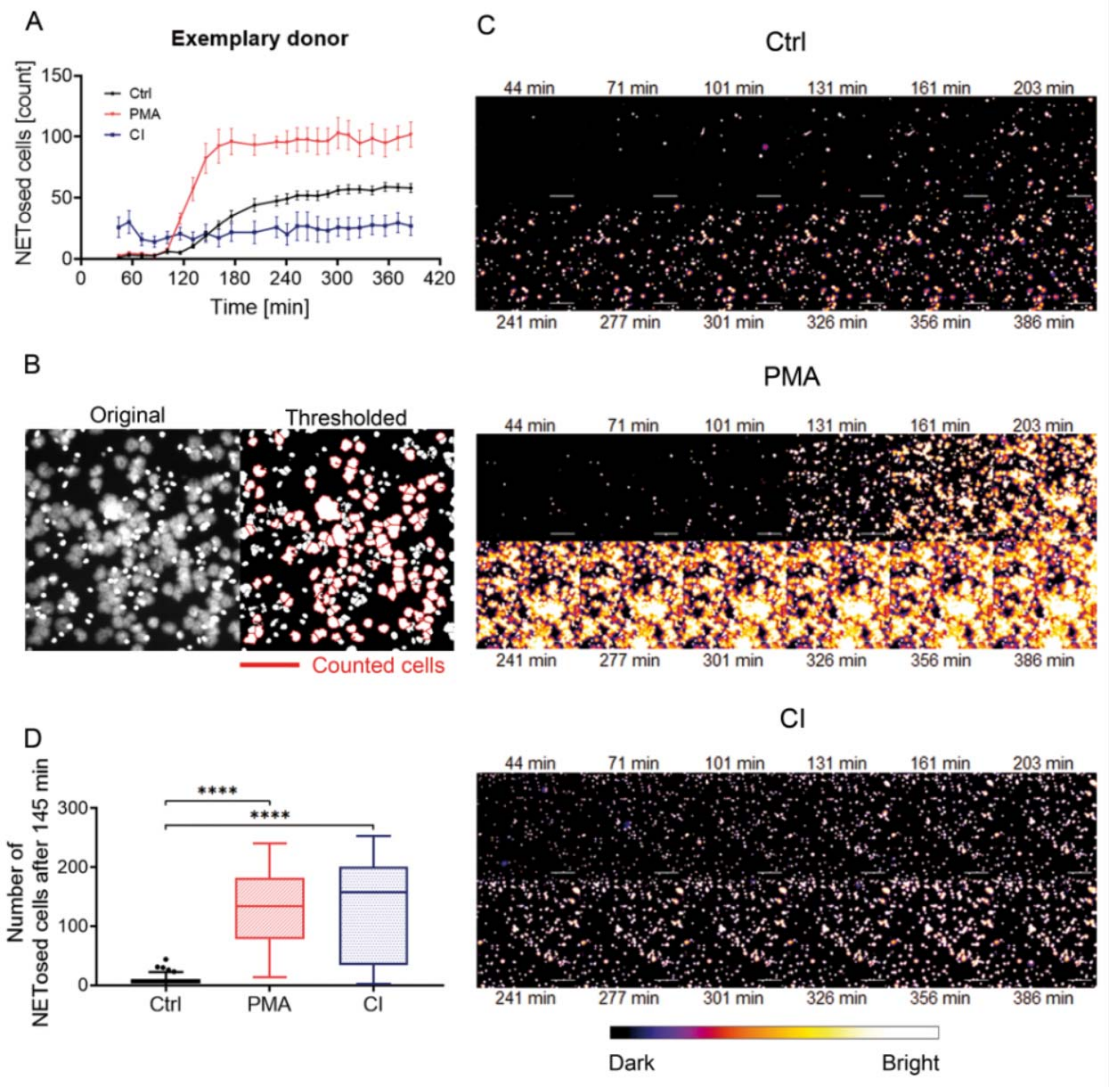
Analysis of live microscopy images obtained from the eSight device. Neutrophils were stained with 1 µM Sytox Green and images taken over a time period of more than 6 h. (A) Time course of counted NETs of an exemplary donor, mean ± SEM. (B) Example image of an automated counting process. Analysis was done by automated counting with ImageJ. ImageJ was advised only to count cells that exceed a certain size (1000 pixel^2^ or 1352 µm^2^). The left image shows the original image from the device, the right image shows the thresholded image with the counted cells marked with a red border. To reduce counting of clusters the “watershed” option in ImageJ was used to separate cells. (C) Time course of cells counted from microscopic images in the green channel (Sytox Green) of an exemplary donor over 6.5 h. Cells were pseudo-colored in “fire” with ImageJ. The bar below the images shows the pseudo-coloring. Scale bar = 200 µm. (D) Analysis of cells undergone NETosis at T=145 min (2 h 25 min). 4 images/condition/timepoint were counted per donor (N=15). ****p<0.001. Ctrl: Control, PMA: phorbol 12-myristate 13-acetate, CI: calcium ionophore A23187.

In addition to the measurement of bio-impedance analysis, the used xCelligence RTCA eSight device allows simultaneous live imaging of the cells which were as well stained with Sytox Green. Over a period of 6.5 h images were taken in fluorescent and bright field channels every 30 - 70 min. Therefore, it is possible to count NETosed cells and to differentiate between NETosed cells (stained (green and) large, pseudo-colored with “fire”, more orange/red) and necrotic cells (stained bright (green) and small, pseudo-colored with “fire” in white). Automatic counting of the fluorescent signals which exceed a certain size (NETosed cells, Figure 3B(Fig. 3)) revealed early activation of CI-stimulated cells which stayed constant over time (Figure 3A(Fig. 3)). PMA stimulated cells showed a steep increase in counted cells after 1.5 h. Control cells increased after 2.5 h showing that also untreated cells start to release NETs after a certain time. Figure 3C(Fig. 3) shows images of the live fluorescence time course (every 30 min) of one exemplary donor pseudo-colored in “fire” to improve the visibility of differences between NETosed (orange/red) and necrotic cells (white).

When analyzing a defined time point (2 h and 25 min / 145 min, Figure 3D(Fig. 3)) PMA stimulation and CI stimulation show a very high count for NETosed cells. CI stimulation showed broader distribution than PMA stimulation, but both stimulations showed a significantly higher count of NETs than control cells.

### Immunofluorescence analysis

**Figure 4:**
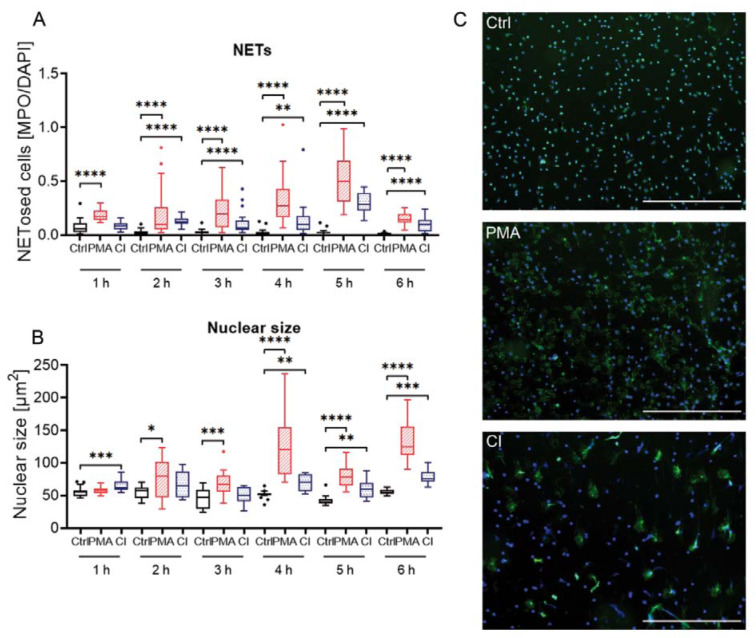
Immunofluorescence analysis of neutrophil stimulation by PMA or CI over a period of 6 h. (A) Analysis of the proportion of cells undergone NETosis by dividing the number of cells stained green (MPO) and exceeding a certain size by total cell number (Hoechst 33342 staining, DAPI channel). (B) Analysis of nuclear size by measurement of Hoechst 33342 staining over a time period of 6 h. N=4. (C) Exemplary overlay images of one donor at 3 h. Scale bar = 400 µm. Blue = Hoechst 33342 staining, green = myeloperoxidase (MPO) staining. Ctrl: Control, PMA: phorbol 12-myristate 13-acetate, CI: calcium ionophore A23187. *p<0.05; **p<0.01; ***p<0.005; ****p<0.001.

To confirm the precedent results, immunofluorescence analysis was done for 4 donors over a period of 6 h with measurement intervals of every hour. DNA was stained with Hoechst 33342 (DAPI channel, blue) and myeloperoxidase (GFP channel, green) by immunofluorescence staining. In the DAPI channel, all cells were counted whereas in GFP channel only cells were counted that exceed a certain size and are not round anymore. By building the ratio of counted cells in the GFP channel (= NETosed cells) to all counted cells the proportion of activated cells can be calculated (Figure 4A(Fig. 4)). Additionally, the nuclear size was determined from the Hoechst 33342 staining (Figure 4B(Fig. 4)). 

The immunofluorescence analysis showed similar results to the other methods. The proportion of NETosed cells (Figure 4A(Fig. 4)) was the highest for PMA treatment but has peaked at 5 h. In some imaged areas, nearly all cells have undergone NETosis (values of nearly 1). For CI treated cells the overall rate remained lower. It had a nearly stable level for 1-4 h and has a bit higher rate at 5 h. The control cells stayed constantly at a low level. The nuclear size of CI treated cells was already significantly higher than the control cells at 1 h (Figure 4B(Fig. 4)). PMA stimulated cells showed a peak of the nuclear size at 4 h. The increase in nuclear size was stronger for PMA treated cells compared to CI treated cells. The nuclear size of control cells presented almost no changes. Optical analysis of the microscopy images revealed that control cells showed the smallest staining area (Figure 4C(Fig. 4), upper image), PMA treated cells had a large distribution of MPO and Hoechst staining (middle panel) and CI treated cells (lower panel) were characterized by larger spots of strong MPO staining but not such high distribution of nuclear staining. An overview of images of one donor for all time points and single channels can be found in Supplementary Figure 3.

## Discussion

Analysis of neutrophil extracellular traps remains a challenge despite the advances that have been made since its discovery. All commonly used techniques have their limitations and are either suitable for one type of analysis (detailed) or the other (many conditions, screening). Here, we tested the usage of a bio-impedance based platform for detection of the release of neutrophil extracellular traps in comparison to immunofluorescence analysis and analysis of released DNA by fluorescent straining (Sytox Green Assay). 

Sytox Green Assay analysis revealed a fast increase in fluorescence by stimulation with CI. In contrast, stimulation with PMA resulted in a much later but therefore much steeper increase in fluorescence which is in agreement with previously published data (Khan and Palaniyar, 2017[22]; Petchakup et al., 2019[39]). Also, when looking at the microscopy images taken during the measurements a difference between the CI and PMA stimulated cells became obvious. PMA stimulated cells showed a cloudy shape whereas some CI stimulated cells demonstrate strong concentrated staining suggesting necrosis of these cells or an early leakiness of the cells due to impaired membrane integrity (de Bont et al., 2018[12]; Hoppenbrouwers et al., 2017[20]). This can be stated as one of the major drawbacks of the Sytox Green Assay: a clear distinction of dead cells and NETosed cells is not possible just from the fluorescent signal (Carmona-Rivera and Kaplan, 2016[10]). A reported problem with basal staining of neutrophils by Sytox Green could not be observed here (Schröter et al., 2013[47]). Despite these disadvantages, Sytox Green Assay remains one of the easiest and fastest assays to detect extracellular DNA released from neutrophils. However, for verification, other methods are required.

Immunofluorescence analysis gives the most detailed analysis of the NETs released from the neutrophils showing the spatial distribution of the chromatin and myeloperoxidase, a protein that plays an important role during the release of NETs (Metzler et al., 2014[33]). The two different stimulations show different distributions of nuclear and MPO staining. The wider distribution for PMA stimulation and the strong spot-related staining for CI stimulation support the proposed different stimulation pathways for PMA and CI (Khan and Palaniyar, 2017[22]). Such a result cannot be drawn from non-imaging methods like Sytox Green Assay, but the bio-impedance measurement also showed differences. For the quantitative analysis of immunofluorescence images, a very defined set-up and steady quality of microscopy images are necessary. For many donors or many time points as we did it in our work, this remains a challenge and is very time-consuming, especially if more conditions shall be investigated. For a detailed analysis of the spatial distribution of proteins (*e.g.;* nuclear translocation, extracellular release, cytosolic localization) or intracellular processes, immunofluorescence analysis remains the most reliable method. If a screening on the base of immunofluorescence is unavoidable, different methods for the quantification exist: analysis of the expansion of chromatin (Neubert et al., 2018[36]), analysis of two different stainings (*e.g.;* H3-Cit and DAPI) (Brinkmann et al., 2012[6]), or comparison of different forms of DNA by threshold settings (Meher et al., 2018[32]) - but all of them include analysis of DNA staining. A combination of two readouts would be recommended here. We used a combination of two stainings (MPO and Hoechst) and additionally an analysis of the nuclear size.

The bio-impedance analysis is a method that allows a more detailed analysis than Sytox Green Assay and faster results than immunofluorescence analysis. Neutrophils bind to surfaces when getting activated (Neubert et al., 2018[36]), which leads to an increase in cell index. In contrast, cell death leads to a reduction in cell index. Here, PMA stimulation leads to a strong increase in cell index, which shows a strong binding of neutrophils to the surface. CI stimulation showed a lower and less intense peak which argues for less strong binding to the surface or faster DNA release. A second, less marked peak expected to come from the binding of NETs to the surface could not be observed. By coating the plates with antibodies, Yu et al. were able to detect different surface binding dynamics of neutrophils by bio-impedance measurements (Yu et al., 2018[57]). Therefore, a better binding of neutrophils and NETs to the used gold-electrode coated plates could be achieved by the coating (*e.g.;* fibrinogen (Healy et al., 2016[19])) of plates. This could facilitate an even more detailed analysis including proposed NETs (Pires et al., 2016[41]). Such results would allow a follow-up of the results from Erpenbeck et al., which showed neutrophils do not need surface binding in response to all stimuli (Erpenbeck et al., 2019[13]).

An increase in cell index and opacity after stimulation of neutrophils was also observed from Petchakup et al., who developed a platform to directly analyze neutrophil activation from blood in a flow device. The increase in cell index could be observed with CI stimulated cells but not with PMA stimulated cells (Petchakup et al., 2019[39]). This is contrary to our results where a defined peak in cell index by stimulation with PMA was observed. An explanation for this could be the observation time which was only 2 hours in the work of Petchakup et al. (2019[39]). At this time point, we observed a maximum cell index in PMA stimulated cells, which indicated towards many attached but not yet fully NETosed cells. Another explanation could be the flow set-up where measurements were carried out only every 30 min and without a surface to bind to. This could lead to a passing over of activation peaks or non-activation due to the lacking surface. 

Another possible application of the bio-impedance measurement would be the monitoring of interaction of neutrophils with other cells as already done for a lot of other immune cell types, *e.g.;* natural killer cells and T-cells (Fasbender and Watzl, 2018[14]; Seidel et al., 2014[48]). This could be of special interest for the interaction with macrophages, which is thought to play a major role in the development and progress of atherosclerosis (Warnatsch et al., 2016[53]). Coatings for stents or implants could be tested which can be advantageous in the prevention of thrombosis or new plaque formation (Labarrere et al., 2020[24]). 

Detailed mathematical modeling of the changes in impedance after neutrophil stimulation with PMA was done by Schröter et al. revealing that measured results are strongly dependent on the composition of the medium and the cell numbers (Schröter et al., 2015[46]). Therefore, such factors have to be closely documented and controlled when using bio-impedance measurements to monitor NET release. Despite these uncertainties, an application of bio-impedance measurement for the analysis of released DNA into wounds is proposed (Schröter et al., 2013[47]) and further development of platforms for analysis on the single-cell level is ongoing (Asphahani et al., 2011[4]).

An advantage of the here tested xCelligence RTCA eSight system is the possibility to directly combine bio-impedance measurements with live-cell imaging. The increase in chromatin size can easily be monitored also in comparison to dead cells which do not show a higher nuclear size. The usage of Sytox Green staining in combination with the bio-impedance measurement enables a more secure analysis of NET release and could even be more detailed when combined with another fluorescent dye (*e.g.* Hoechst to stain all nuclei, three channels are possible in total). The combination of those two techniques makes it possible to show the dynamics of early events (bio-impedance) as well as later events (DNA release by Sytox Green staining). So the bio-impedance would allow monitoring dynamics before the so-called “point of no return” and the live imaging of Sytox Green staining the passive events afterwards (Neubert et al., 2018[36]).

**Figure 5:**
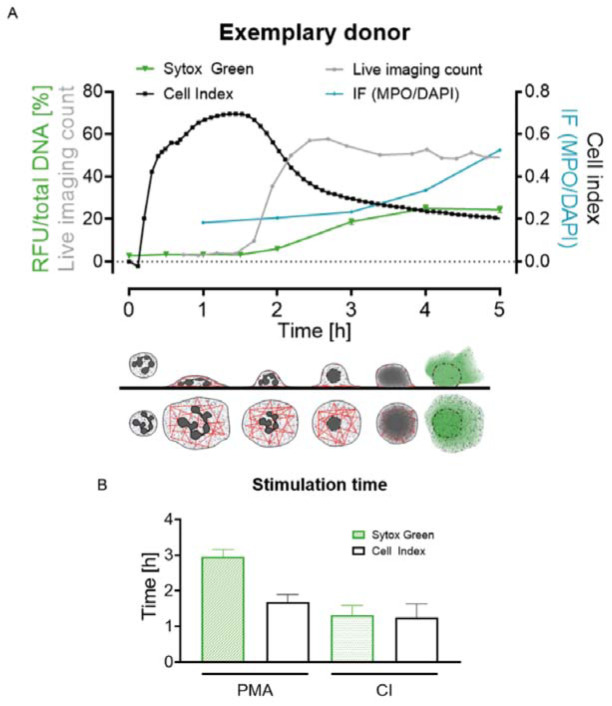
Summary and schematic overview of cell index and fluorescence change during the NET release of neutrophils. (A) Data of all four made analyses (Sytox Green Assay, bio-impedance (cell index) measurement, count of live cell imaging, immunofluorescence) of one donor for 6 h. Lower panel shows assumed cellular events schematically from the side and from the top view (gray = nucleus/DNA, red = Actin, green = fluorescently marked DNA when released). (B) Comparison of peak times (bio-impedance analysis, white bars) and half-maximal stimulation time (Sytox Green Assay, green bars) of PMA and CI stimulated cells, N=15. Ctrl: Control, PMA: phorbol 12-myristate 13-acetate, CI: calcium ionophore A23187, RFU: relative fluorescence unit, IF: immunofluorescence.

When combining the data from the four different assays for PMA stimulation (Figure 5A(Fig. 5)) a more detailed time-course analysis by the bio-impedance analysis and the Sytox Green Assay is revealed. The bio-impedance analysis shows a peak very early compared to Sytox Green Assay and immunofluorescence analysis. If comparing the peak times of Sytox Green Assay (time of half-maximal stimulation) and bio-impedance measurement (peak time) in detail (Figure 5B(Fig. 5)) for PMA stimulation the peak time is much later in Sytox Green Assay than in the bio-impedance analysis. For CI stimulation the peak time is similar, most likely due to the fast dynamics by the stimulation with calcium ionophore thus no comparing overview like for PMA (Figure 5A(Fig. 5)) was done.

In conclusion, impedance measurement allows for label-free analysis, detailed time-course analysis, and additional staining improves the validity of measurements but is not generally necessary. For a total analysis of NET release, a combination of different methods (screening and imaging-based methods) is still inevitable but bio-impedance measurements could be another part in the repertoire of neutrophil extracellular trap analysis and participate in the ongoing attempt to fully understand the process.

## Funding

C.L. is supported by the Studienstiftung des deutschen Volkes.

## Conflict of interest

The authors declare that they have no conflict of interest.

## Acknowledgements

We thank Dr. Andreas Friese from Omni Life Sciences for the opportunity to test the xCelligence device. We thank Dr. Markus Burkhard and Christian Leischner for the assistance during measurements with the xCelligence device.

## Supplementary Material

Supplementary material
