# Supplementary material for: Bio-impedance measurement allows displaying the early stages of neutrophil extracellular traps
Source: EXCLI J. 2020 Nov 5;19:1481–95. doi: 10.17179/excli2020-2868 (PMC7689246; doi:10.17179/excli2020-2868)
Supplement: Supplementary material [file EXCLI-19-1481-s-001.pdf]

EXCLI Journal 2020;19:1481-1495 – ISSN 1611-2156
Received: September 04, 2020, accepted: October 29, 2020, published: November 05, 2020

Supplementary material to:

BIO-IMPEDANCE MEASUREMENT ALLOWS DISPLAYING THE EARLY STAGES OF NEUTROPHIL EXTRACELLULAR TRAPS

Caren Linnemann[1], Sascha Venturelli[2,3], Franziska Konrad[4], Andreas K. Nussler[1], Sabrina Ehnert[1*]

[1] Siegfried Weller Institute for Trauma Research, BG Unfallklinik Tuebingen, Eberhard Karls Universität Tuebingen, Tuebingen, Germany
[2] Institute of Physiology, Department of Vegetative and Clinical Physiology, University Hospital Tuebingen, Tuebingen, Germany
[3] Institute of Nutritional Sciences, Department of Nutritional Biochemistry, University of Hohenheim, Stuttgart, Germany
[4] Department of Anesthesiology and Intensive Care Medicine, University Hospital of Tuebingen, Tuebingen, Germany

* **Corresponding author:** Dr. Sabrina Ehnert, Siegfried Weller Institute for Trauma Research at BG Unfallklinik Tuebingen, Eberhard Karls Universität Tuebingen, Schnarrenbergstr. 95, 72076 Tuebingen, Germany; Tel.: +497071 606 1065, Fax.: +49 70 71 606 1978; E-mail: sabrina.ehnert@gmail.com;
ORCID: https://orcid.org/0000-0003-4347-1702

http://dx.doi.org/10.17179/excli2020-2868

This is an Open Access article distributed under the terms of the Creative Commons Attribution License (http://creativecommons.org/licenses/by/4.0/).

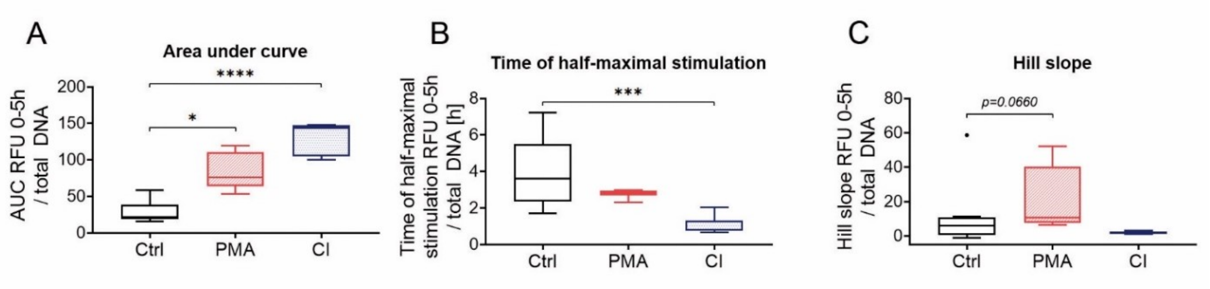

Supplementary Figure 1: Sytox Green Assay normalized to total DNA values. Neutrophils were stained with 1 µM Sytox Green and fluorescence measured over a time of 5 h. Different analysis methods were used to show neutrophil activation by different stimuli. (**A**) Analysis of DNA release by measurement of area under curve. (**B**) Analysis of stimulation time by determination of half-maximal stimulation time. (**C**) Analysis of strength of stimulation by measurement of hill slope. All measured fluorescence data were normalized to total DNA values determined by 1 % Triton-X-100 treated cells. N=15, n=3. Statistical analysis was done by Kruskal-Wallis test followed by Dunn's correction for multiple comparisons. Ctrl: Control, PMA: phorbol 12-myristate 13-acetate, CI: calcium ionophore A23187, AUC: Area under curve, RFU: relative fluorescence unit.

EXCLI Journal 2020;19:1481-1495 – ISSN 1611-2156
Received: September 04, 2020, accepted: October 29, 2020, published: November 05, 2020

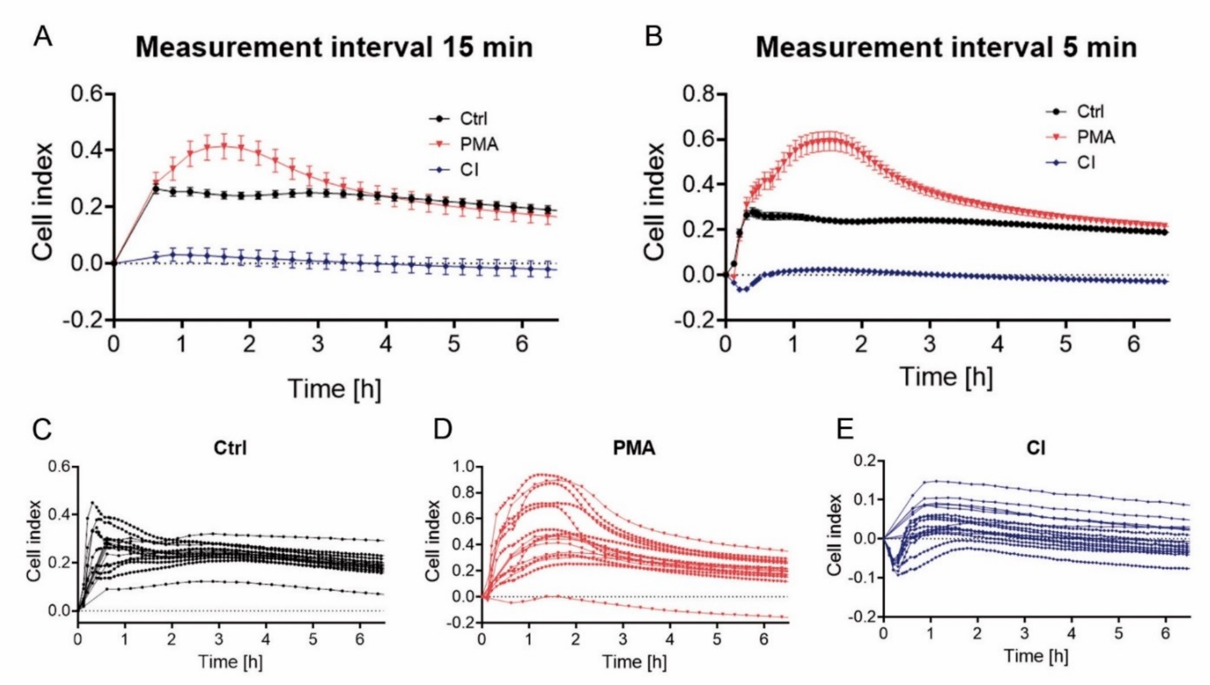

Supplementary Figure 2: Raw data of bio-impedance measurement for the activation of neutrophils. Neutrophils were seeded in eSight E-Plate VIEW 96 and bio-impedance measured for over 6 h. (**A**) Mean values of 8 donors with a measurement interval of 15 min. (**B**) Mean values of 8 donors with a measurement interval of 5 min. (**C**) Values of unstimulated neutrophils of all 15 donors. (**D**) Values of PMA-stimulated neutrophils of all 15 donors. (**E**) Values of CI-stimulated neutrophils of all 15 donors. Mean ± SEM. Ctrl: Control, PMA: phorbol 12-myristate 13-acetate, CI: calcium ionophore A23187.

EXCLI Journal 2020;19:1481-1495 – ISSN 1611-2156
Received: September 04, 2020, accepted: October 29, 2020, published: November 05, 2020

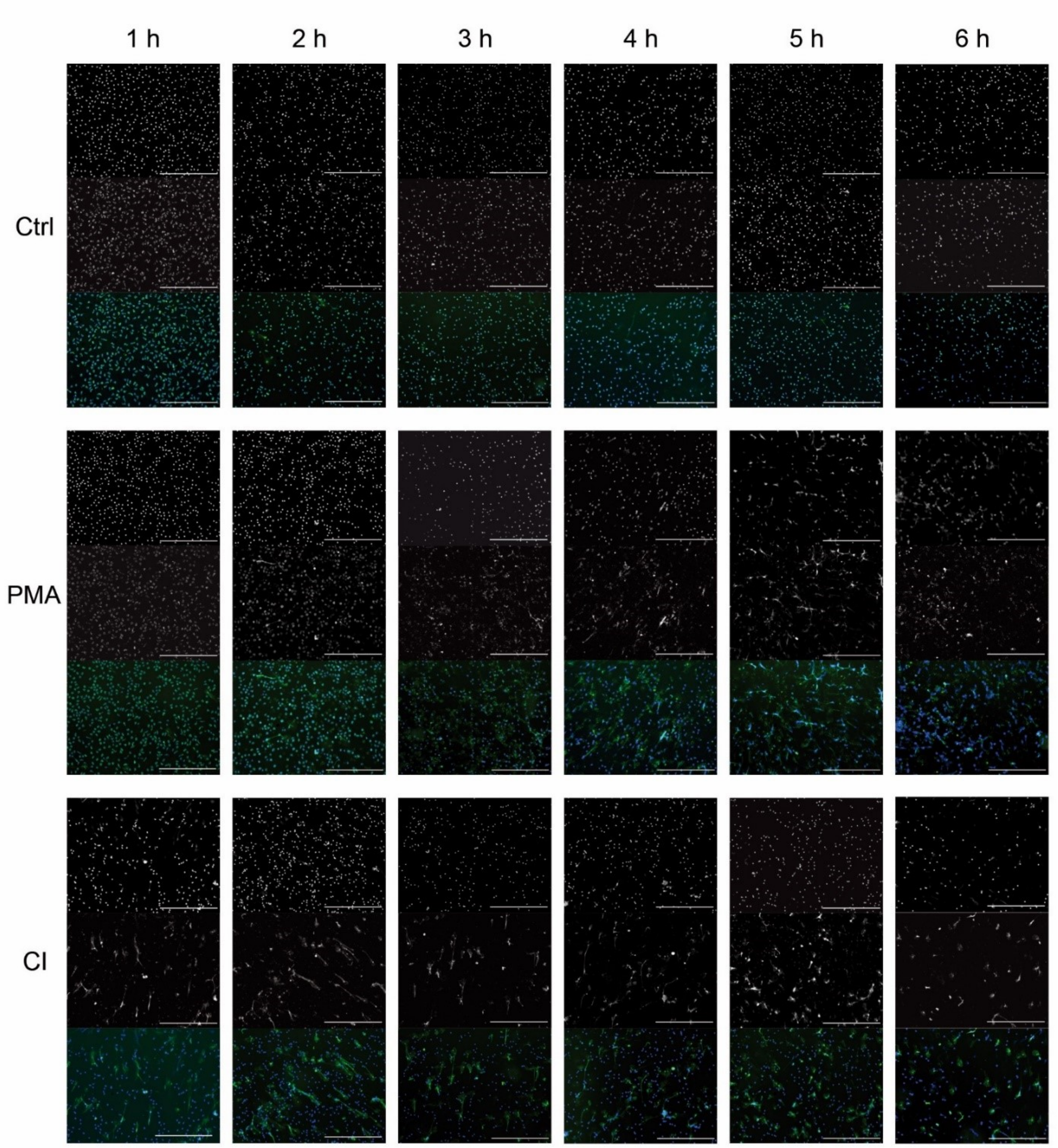

Supplementary Figure 3: Immunofluorescence images from exemplary donor over time-course of 1-6 h. Example images of one donor over the time course from 1 h to 6 h. Upper panels display nuclear staining with Hoechst 33342 in gray scale, middle panels show myeloperoxidase staining in gray scale. Lower panels display overlay images in color. Scale bar = 400 µm. Blue = Hoechst 33342 (nuclear) staining, green = myeloperoxidase (MPO) staining. Cells were fixed with 4 % formaldehyde at defined time points. Ctrl: Control, PMA: phorbol 12-myristate 13-acetate, CI: calcium ionophore A23187.

EXCLI Journal 2020;19:1481-1495 – ISSN 1611-2156
Received: September 04, 2020, accepted: October 29, 2020, published: November 05, 2020

Supplementary Table 1: Quality of curve fit analysis of PMA-peak modulation of cell index measurements

Donor	Data points	Adjusted R squared	Standard deviation of residuals
**#1**	36	0.6767	0.02598
**#2**		0.6802	0.03737
**#3**		0.8066	0.01908
**#4**		0.6311	0.05734
**#5**		0.8836	0.01447
**#6**		0.6363	0.03664
**#7**		0.8313	0.02138
**#8**		0.3688	0.02199
**#9**	96	0.9534	0.009359
**#10**		0.9261	0.02359
**#11**		0.9482	0.01005
**#12**		0.9373	0.01229
**#13**		0.94	0.0282
**#14**		0.9632	0.01951
**#15**		0.935	0.03064
**#17**		0.5936	0.03281
General formula: $Y=B_0 + B_1X + B_2X^2$			
